# Economic evaluations of interventions to reduce neonatal morbidity and mortality: a review of the evidence in LMICs and its implications for South Africa

**DOI:** 10.1186/s12962-015-0049-5

**Published:** 2016-01-26

**Authors:** Mandy Maredza, Lumbwe Chola, Karen Hofman

**Affiliations:** Priority Cost-Effective Lessons for Systems Strengthening-South Africa (PRICELESS SA), Medical Research Council/Wits Rural Public Health and Health Transition Research Unit (Agincourt), Johannesburg, South Africa; School of Public Health, Faculty of Health Sciences, University of the Witwatersrand, Johannesburg, South Africa

**Keywords:** Economic evaluation, Cost-effectiveness analysis, Literature review, Neonatal health, Low and middle-income countries

## Abstract

**Background:**

Newborn mortality, comprising a third of all under-5 deaths, has hardly changed in low and middle income countries (LMICs) including South Africa over the past decade. To attain the MDG 4 target, greater emphasis must be placed on wide-scale implementation of proven, cost-effective interventions. This paper reviews economic evidence on effective neonatal health interventions in LMICs from 2000–2013; documents lessons for South African policy on neonatal health; and identifies gaps and areas for future research.

**Methods:**

A narrative review was performed in leading public health databases for full economic evaluations conducted between 2000 and 2013. Data extraction from the articles included in the review was guided by the Consolidated Health Economic Evaluation Reporting Standards (CHEERS) checklist, and the quality of the included economic evaluations was assessed using the Quality of Health Economics Studies Instrument (QHES).

**Results:**

Twenty-seven economic evaluations were identified, from South East Asia and sub-Saharan Africa, with those from sub-Saharan Africa primarily focused on HIV/AIDS. Packages of care to prevent neonatal mortality were more cost-effective than vertical interventions. A wide variability in methodological approaches challenges the comparability of study results between countries. In South Africa, there is limited cost-effectiveness evidence for the interventions proposed by the National Perinatal Morbidity and Mortality Committee.

**Conclusions:**

Neonatal strategies have a strong health system focus but this review suggests that strengthening community care could be an additional component for averting neonatal deaths. While some evidence exists, having a more complete understanding of how to most effectively deploy scarce resources for neonatal health in South Africa in the post-2015 era is essential.

**Electronic supplementary material:**

The online version of this article (doi:10.1186/s12962-015-0049-5) contains supplementary material, which is available to authorized users.

## Background

International child health policy in the last decade has been largely shaped by millennium development goal 4 (MDG 4), to reduce under-5 mortality by two-thirds between 1990 and 2015. Accelerated declines have been observed in developed regions with under-5 mortality reducing by more than 50 % [1]. However, fewer than 25 % of 137 low and middle income countries (LMICs) are on track to reach the MDG 4 target [2]. Persistently high neonatal mortality, which now accounts for a greater proportion of global child deaths (40 %) than in 1990, is one of the contributing factors to this slower than anticipated progress [3].

In South Africa, under-5 mortality reduced by 40 % from 2006 to 2011 largely due to the successful scale up of prevention of mother to child transmission (PMTCT) of HIV [4]. Yet, newborn mortality remains stagnant at a level of 14–20 deaths per 1000 live births [3, 5]. This is despite the fact that 80 % of births are in facilities [6], and there is a national policy of free maternal and child healthcare at public facilities (enacted since 1996) [7]. Health care spending is one of the highest in Africa, albeit with little return on investment [8]. The high neonatal mortality rate is associated with poor quality care, sub-optimal adherence to guidelines, delays in seeking antenatal care, inadequate inter-facility transport for emergency obstetric care and inadequate postnatal care [9].

To accelerate progress beyond 2015 and as South Africa moves towards universal health coverage, we need to understand which priority interventions can be scaled up to save newborn lives, and the related resource implications. Economic data on essential newborn interventions is limited in South Africa, but several studies have been undertaken in other LMICs [10–12].

Policy makers in South Africa are increasingly aware of the need to justify health policy decisions on the basis of both effectiveness and costs of interventions. Previous work done by the authors on the cost and impact of reducing sodium content in high salt foods [13] led to the government’s drafting of policy on salt regulation, setting targets for 2016 and 2018 [14]. More recently, work on identifying the costs and impact of essential interventions for maternal and child health has been adopted by the National Department of Health as priority interventions for preventing additional deaths of mothers and children in the countdown to the Millennium Development Goals [15]. Follow-up work focused on family planning, childhood diarrhoea and stillbirths, provided insight on the impact and costs of scaling up interventions to reduce maternal, newborn and child mortality in South Africa [16–18]. Despite these efforts, more still needs to be done to generate information that is necessary for evidence based decision making in South Africa.

This review of economic evaluations of antenatal, intrapartum and postnatal interventions aims to: (1) identify the key interventions for which economic data exist in LMICs, (2) assess the relevance of the available data to South Africa; (3) identify gaps in knowledge and priority areas for future research; and (4) to assess the quality of the economic evaluations included in the review. This information could be useful for South Africa in its process of implementing universal health coverage.

## Methods

A literature review was conducted by the first and second authors between October and December 2013. We used the approach by Glanville et al. (2009) to guide the literature search [19]. Specific databases which collect economic evaluations including the National Health Service Economic Evaluation Database (NHS EED), Cochrane and the Paediatric Economic Evaluation Database (PEED) were searched first. This was followed by top-up searches in large databases namely Medline, Embase and the WHO Global Health Library. We elected this approach as evidence indicates that commonly used search filters for retrieving economic evaluation articles in large biomedical databases such as Medline and Embase suffer from low precision, returning many irrelevant records [19]. We used a combination of search strategies in each of the databases and these are shown in Additional file 1.

Abstracts of each identified article were assessed for eligibility using the following inclusion criteria:

- Articles were published in English in peer-reviewed journals (2000–2013);
- The study setting was LMICs;
- Full economic evaluations, which compared the costs and outcomes of at least 2 alternative strategies;
- Studies used primary or secondary data;
- Original articles published in international journals; and
- Published prior to December 2013.

Articles that did not fit this criterion were excluded. The period 2008–2013 was chosen for articles focusing on PMTCT because of the rapid advancement and changes in PMTCT protocols, and since many of the interventions assessed before this period are outdated. Assessment for article inclusion was done by the first and second authors, with disagreements resolved through subsequent discussions. The inter-rater agreement for selection was 96 %.

### Extraction of information

The Consolidated Health Economic Evaluation Reporting Standards (CHEERS) 24 item checklist was used to extract information from each of the studies included [20]. The CHEERS checklist was designed to be an aid to researchers reporting economic evaluations. It suggests standard items that should be included in an economic evaluation to facilitate standard reporting. We used CHEERS to extract economic information from identified articles, including the time horizon, discount rate, choice of health outcomes, measure of effectiveness, incremental costs and outcomes, study perspective and comparators. Other information that was extracted included the year of publication, authors, affiliation of the first author, type of journal and country in which the study was undertaken. Data extraction was independently done by the first and second authors. Inter-rater agreement for selection was 85 %. Any disagreements were resolved through subsequent discussions.

An assessment of quality was made using the Quality of Health Economics Studies Instrument (QHES), a validated quality-scoring instrument (score range = 0–100; >75 = high quality) [21]. The QHES is a practical quantitative tool for appraising the quality of cost-effectiveness studies. Using this tool, studies are graded on whether they provide relevant information that is standard to reporting in economic evaluations, such as the statement of clear objectives, the study perspective and its justification, handling of uncertainty and choice of the economic model. The checklist gives weighting scores to different quality indicators as shown in Table 1. The quality scoring was done independently by the first and second authors, and then compared for agreement. Disagreements were resolved through subsequent discussions. The agreement on scoring was 79 %.

**Table 1:** The quality of health economic studies (QHES) instrument

	Questions	Weight
1	Was the study objective presented in a clear, specific, and measurable manner?	7
2	Were the perspective of the analysis (societal, third-party payer, etc.) and reason for its selection stated	4
3	Were variable estimates used in the analysis from the best available source (i.e. randomized control trial—best, expert opinion—worst)?	8
4	If estimates came from a subgroup analysis, were the groups prespecified at the beginning of the study?	1
5	Was uncertainty handled by: (1) statistical analysis to address random events; (2) sensitivity analysis to cover a range of assumptions?	9
6	Was incremental analysis performed between alternatives for resources and costs?	6
7	Was the methodology for data abstraction (including the value of health states and other benefits) stated?	5
8	Did the analytic horizon allow time for all relevant and important outcomes? Were benefits and cost that went beyond 1 year discounted and a justification given for the discount rate?	7
9	Was the measurement of costs appropriate and the methodology for the estimation of quantities and unit costs clearly described?	8
10	Were the primary outcome measure(s) for the economic evaluation clearly stated and were the major short-term, long-term, and negative outcomes included?	6
11	Were the health outcomes measures/scales valid and reliable? If previously tested, valid and reliable measures were not available, was justification given for the measures/scale used?	7
12	Were the economic model (including structure), study methods and analysis, and the components of the numerator and denominator displayed in a clear transparent manner?	8
13	Were the choice of economic model, main assumptions and limitations of the study stated and justified?	7
14	Did the author(s) explicitly discuss direction and magnitude of potential biases?	6
15	Were the conclusion/recommendations of the study justified and based on the study results?	8
16	Was there a statement disclosing the source of funding for the study?	3

Cost-effectiveness was first reported as stated by authors and in this paper, cost-effectiveness also includes cost-utility and cost-minimization. We standardised costs to international dollars using the approach used by Prost et al. (2013), and report all costs and cost-effectiveness results in cost per 2013 international dollars (denoted as I$) [22]. The reported United States Dollar (US$) were converted into local currency using the exchange rates for the cost year, adjusted for inflation to calculate the cost in the current year of analysis, then reconverted to US$ Purchasing Power Parity (PPP) or international dollars. Currency conversion factors on OANDA were used in this study [23]. For regional analyses (e.g. sub-Saharan Africa), where costs were reported in US$, we adjusted for inflation using the regional GDP deflator [24]. We assumed that the cost year was the year in which the intervention was conducted, if this was not specified in the article. Cost-effectiveness thresholds were based on WHO recommended methods, i.e., each intervention was classified as (a) highly cost-effective if it averted a year of life lost or if it averted a DALY for less than the national gross domestic product (GDP) per capita (in I$), (b) cost effective if 1–3 times GDP per capita, and (c) not cost-effective if greater than 3 times the GDP per capita.

## Results

**Fig. 1:**
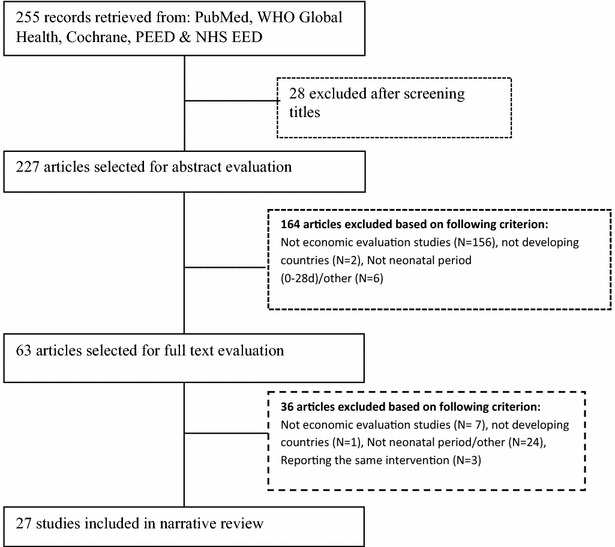
Overview of the literature search, inclusion and exclusion criteria

A total of 255 articles were identified, of which 28 were excluded in the initial screening, because they did not meet the basic inclusion criteria (Fig. 1). A further 164 articles were excluded because they were not economic evaluations, or were economic evaluations not done in low and middle income countries, or were of children not in the neonatal period. A total of 63 articles were selected for full text evaluation, of which 37 were excluded because they did not meet one or more of the inclusion criteria. The remaining 27 articles spanning a 13 year period (2000–2013) were included in this review (Additional file 2: Table S1).

### Characteristics of included studies

Of the included articles, 5 (19 %) were published before 2007 and 22 (81 %) between 2007 and 2013 (Table 2). The studies were conducted in Africa (59 %), Asia (30 %) or included multiple analyses countries from various LMICS. The majority of the studies assessed preventive interventions (70 %), while others were of curative (7 %) or diagnostic (7 %) interventions. About 48 % of the studies followed a modelling approach, 30 % were based on randomized controlled trials and a further 22 % used observational studies. More than half (52 %) of the studies reviewed were cost-effectiveness analyses (i.e. the outcomes used were intermediate measures or natural units, deaths prevented or cases detected). The rest (48 %) were cost-utility analyses (i.e. they used composite outcome measures such as disability-adjusted life years or quality-adjusted life years). Primary data were used in 48 % of the studies.

**Table 2:** Characteristics of studies included in the review

Characteristic	N = 27	Percentage
Year of publication		
Before 2007	5	19
After 2007	22	81
Region		
Africa	16	59
Asia	8	30
LMICS	3	11
Intervention type		
Preventive	19	70
Curative	2	7
Preventive/curative	4	15
Diagnostic/screening	2	7
Study design		
Randomized controlled trials	8	30
Observational	6	22
Modelling	13	48
Study type		
Cost-utility analysis	13	48
Cost-effectiveness analysis	14	52
Type of data used		
Primary	13	48
Secondary	14	52

### Quality of included studies

The studies included in the literature review were of variable quality (Table 3). Fourteen (52 %) of the studies were graded high, 12 (44 %) were thought to be fair and 1 (4 %) was poor.

**Table 3:** Quality index scores for studies included in the review

	Study	Quality index decision based on % score
1	Adam et al. (2005)	Fair
2	Borghi et al. (2005)	High
3	Manasyan et al. (2011)	Fair
4	Bang et al. (2005)	Fair
5	Tripathy et al. (2004)	Fair
6	Lewycka et al. (2013)	Fair
7	Fottrell et al. (in Prost et al. 2013)	Fair
8	LeFevre et al. (2013)	High
9	Owusu-Edusei et al. (2011)	High
10	Sicuri et al. (2010)	High
11	Sabin et al. (2005)	High
12	Sayed et al. (2008)	Fair
13	Halperin et al. (2009)	Fair
14	John et al. (2008)	Fair
15	Robberstad and Ovjen-Olsen (2010)	High
16	Orlando et al. (2010)	Fair
17	Shah et al. (2011)	High
18	Maredza et al. (2013)	High
19	Binagwaho et al. (2013)	High
20	Hung et al. (2011)	High
21	Bomela et al. (2001)	Poor
22	Vickerman et al. (2006)	Fair
23	Hong et al. (2010)	High
24	Hounton et al. (2009)	High
25	Huang et al. (2012)	High
26	Darmstadt et al. (2007)	Fair
27	Fasawe et al. (2013)	High

High = 75–100 %; fair = 50–74 %; poor = 25–49 %

The proportion of studies that met the criteria for reporting of economic evaluations used in the quality index tool is given in Table 4. Economic evaluation was not the primary objective in all the studies, hence some (24 %) did not clearly articulate the objectives of their evaluations. The study perspective was stated in 76 % of the articles, though some of these did not justify the choice of perspectives. The health care provider perspective was the most common. Sensitivity analyses were done in 66 % of the studies. Sensitivity analyses methods varied with some studies using one-way [10, 11, 25, 26, 27], others using probabilistic tests [28–30] and a few using both [31]. Incremental analysis was performed in 66 % of the studies, and 62 % clearly showed the costing methods. About 59 % of the papers met the criteria on discounting. None of the articles, however, provided a justification for the discount rate. The economic model used, including the structure, was clearly presented in 9 (31 %) of the studies and 6 (21 %) justified the choice of the economic model. Only 34 % of the studies disclosed their funding source.

**Table 4:** Proportion of studies that met the selected criteria for grading economic evaluations

	Questions	% (N = 27)
1	Was the study objective presented in a clear, specific, and measurable manner?	76
2	Were the perspective of the analysis (societal, third-party payer, etc.) and reason for its selection stated	76
5	Was uncertainty handled by: (1) statistical analysis to address random events; (2) sensitivity analysis to cover a range of assumptions?	66
6	Was incremental analysis performed between alternatives for resources and costs?	65
7	Was the methodology for data abstraction (including the value of health states and other benefits) stated?	76
8	Did the analytic horizon allow time for all relevant and important outcomes? Were benefits and cost that went beyond 1 year discounted and a justification given for the discount rate?	59
9	Was the measurement of costs appropriate and the methodology for the estimation of quantities and unit costs clearly described?	62
10	Were the primary outcome measure(s) for the economic evaluation clearly stated and were the major short-term, long-term, and negative outcomes included?	90
11	Were the health outcomes measures/scales valid and reliable? If previously tested, valid and reliable measures were not available, was justification given for the measures/scale used?	86
12	Were the economic model (including structure), study methods and analysis, and the components of the numerator and denominator displayed in a clear transparent manner?	31
13	Were the choice of economic model, main assumptions and limitations of the study stated and justified?	21
14	Did the author(s) explicitly discuss direction and magnitude of potential biases?	86
15	Were the conclusion/recommendations of the study justified and based on the study results?	93
16	Was there a statement disclosing the source of funding for the study?	34

Criteria 3 and 4 not included in this table. See Table 1 for full list of criteria

### Summary of the evidence in the included studies

To summarize the evidence, the studies are divided into 4 groups according to interventions assessed: community care packages, facility based neonatal care packages, PMTCT programmes and other vertical interventions.

### Economic evaluation of community based interventions

There were ten studies that investigated the economic impact of community care packages on neonatal morbidity and/or mortality (Table 5). Five were based on cluster randomized trials of women’s participatory groups, and of these, four were conducted in rural settings in Asia. In these studies, women’s group facilitators (who were not health workers) received 7–11 days of training and moderated community group meetings. During these meetings, women designed and implemented strategies to address obstetric and perinatal problems relevant to their setting. In both intervention and control areas, health system strengthening activities were conducted, which included training of health workers in essential newborn care and safe motherhood. In addition, neonatal resuscitation equipment was donated to all facilities [32]. The success of the interventions was attributed to several factors including clean delivery practices at home (handwashing), immediate postnatal care [11, 33], increased uptake of antenatal care (15), increased uptake of institutional deliveries (15), increased care seeking for neonates [11] and exclusive breastfeeding for at least 6 weeks [33] to 6 months [34] of life.

**Table 5:** Cost-effectiveness ratios for the participatory women’s groups’ interventions, community care packages, and facility based packages for neonatal health in LMICs (in 2013 International dollars)

Study	Cost of women’s group intervention per newborn life saved (with health systems strengthening)	Cost of women’s group intervention per year of life lost averted	Gross domestic product per person (current international dollar)
Women’s group interventions			
Tripathy et al. (2010) (India)	2022 (2908)	73 (106)	5418
Lewycka et al. (2013) (Malawi)	11,240	290	780
Fottrell et al. (2013) (in Prost et al. 2013) (Bangladesh)	45,420	1490^a^	2948
Borghi et al. (2005) (Nepal)	N/A	428 (509)	2244
Community based packages			
Sabin et al. (2012) (Rural Zambia)	2230	205	3925.50
LeFevre et al. (2013) (Bangladesh)—neonatal care package	6740	240	2948
Bang et al. (2005) (Gadchiroli, India)	N/A	16	5418
Lewycka et al. (2013) (Malawi)	2584	N/A	780
Facility based package			
Manasyan et al. (2011) (Urban Zambia)—early neonatal care package	264	7	3925

The numbers in parenthesis represent the cost-effectiveness when health systems strengthening initiatives were included

^a^Reported DALYs not YLL

The main costs included in each trial were the economic costs of setting up and running the women’s group intervention. Costs related to health service strengthening were also reported in all studies. However, only one study provided input parameters for the cost analysis [32]. In this study, personnel costs were the biggest cost-drivers, accounting for 70 % of total costs. Incremental costs per year of life saved differed across settings but were lower than the national GDP per capita in all cases. Even though life years saved (LYS) was a common outcome measure, in one trial LYS included both maternal and infant deaths averted [34] whilst the rest only included neonatal deaths.

Potential effect modifiers differed from one trial to another. In Nepal, women’s groups collaborated with female community health workers who had been trained in essential newborn care [11]. In Jharkhand, India, pre-existing women’s groups that were involved in savings and credit schemes formed some of the groups that subsequently participated in the trial [33]. In addition, health committees were formed that allowed participants to express opinions about the management and the design of local health services, thereby engaging more closely with the public health system. In Malawi, trial membership was expanded to men residing within the study setting [34].

For the remaining five studies, costs and impact of variant community care packages were assessed. Two of these studies conducted in rural Zambia and Malawi, used peer counsellors and traditional births attendants (TBAs) to deliver the interventions [29, 34]. In the Lufwanyama Neonatal Survival Study (LUNESP) in Zambia, traditional birth attendants (TBAs) were trained for 4 days to perform neonatal resuscitation using resuscitation masks, administer antibiotics and to promptly refer neonates with sepsis to health facilities [29]. One to two refresher trainings were given every 3–4 months. In Muchinji district (Malawi), peer counsellors were trained to conduct pregnancy surveillance, health education on exclusive breastfeeding, infant care, immunizations, PMTCT and family planning [34]. Incremental cost-effectiveness ratios for the TBA and peer-counsellor driven interventions were I$74 and I$60 per DALY prevented, respectively, and were much lower than the GDP per capita of Zambia and Malawi, respectively. Detailed cost analyses were available for one study and indicated that personnel costs, arising from the extensive training accounted for 60 % of total costs [29]. The costs included both direct (provider) costs and indirect costs incurred due to healthcare seeking.

The other three studies were based on interventions implemented in India [32] and Bangladesh [28]. In India, a home based package was developed to equip village health workers (VHWs) in resuscitation techniques and early management of danger signs such as feeding problems, and breathing difficulties. Furthermore VHWs were trained to encourage mothers to practice kangaroo mother care, early and exclusive breastfeeding and to offer postnatal care [32]. Though detailed information was not available, the cost analyses from a provider perspective indicated that personnel costs were the biggest cost drivers accounting for 70 % of total costs. The intervention was highly cost-effective at I$14/DALY averted and this high cost-effectiveness could be partially attributed to inclusion of still births (not just neonatal deaths) in calculation of DALYs averted.

In a similar initiative in Bangladesh, community health workers (CHWs) made two home visits to pregnant women at 12–16 and 32–34 weeks of gestation, trained in birth and neonatal care preparedness and conducted three postnatal visits at 1, 3 and 7 days after birth. Furthermore health systems strengthening activities were carried out, which included training key staff from local health centres in maternal and neonatal health and provision of essential drugs. Similar to findings from other trials, personnel costs accounted for the largest proportion of the recurrent costs (55 %), followed by transportation. Relative to the control arm, the intervention cost was I$211 per additional DALY averted [28]. The cost-effectiveness ratio was much lower than GDP per capita of Bangladesh in 2011, which was I$1700.

Contextual factors potentially impacted on effect and cost-effectiveness of interventions. In two trials, training was conducted by a foreign based neonatologist with support from local midwives and facilitators [12, 29]. Substantial training and retraining was a critical component in the trials, with the LUNESP intervention [29] offering 2–3 refresher trainings every 2–3 months. In all studies, interventions were implemented by Non-Governmental Organizations (NGOs), with a new workforce introduced that focused solely on neonatal and/or maternal health. Furthermore, in India, the NGO that implemented the trial had established a service base whilst running a surveillance system in the area prior to the study and had earned the trust of the local population [32]. Performance-linked remuneration offered to village health workers was another important potential effect modifier in the trials [32] but details regarding the actual unit costs for this were not available.

### Facility-based neonatal care packages

One study investigated the cost-effectiveness of re-training midwives in essential newborn care (ENC) in first level urban health facilities in Zambia [12]. The ENC package covered resuscitation techniques, importance of kangaroo mother care, early and exclusive breastfeeding and early detection and management of danger signs. This intervention was highly cost-effective (I$5.71/DALY averted) in comparison to the rest of community care packages studied. However, only transport costs of the foreign based master trainer were included and not salary costs as the trainer worked on a voluntary basis.

### Economic evaluation of vertical interventions

The vertical intervention studies focused on diverse topics ranging from nutritional supplementation [35], malaria [36], syphilis [26, 27, 30], neonatal asphyxia, bilateral congenital hearing loss [37], hepatitis B [38] and the role of community health workers in home deliveries [32] (Table 2).

Cost-effectiveness ratios were reported as cost per deaths averted, DALYs averted, HIV infections prevented, newborn lives saved, case fatality rates and years lived with disability. In one study, cost-benefit ratios were used [35]. It is difficult to compare cost-effectiveness results as the outcomes measured differed across studies. Even where similar outcomes were measured such as DALYs, valuing techniques differed. In some studies the YLL (premature mortality) included stillbirths, neonatal deaths and miscarriages [26] whilst others only included neonatal deaths. Notwithstanding these limitations most of the interventions studied were considered cost-effective in the study settings due to the potentially large number of life years gained by reducing neonatal deaths.

Amongst the studies that reported cost/DALY, intervention cost-effectiveness ranged from I$2/DALY averted for preventive malaria treatment during pregnancy to I$326/DALY averted for congenital syphilis screening [26]. On average every I$21 saved a neonate from asphyxia when a simple bag-and-mask was used for resuscitation [32]. Task-shifting of emergency obstetric care to general practitioners cost I$200 per decrease in neonatal case fatality [10]. Other interventions that were considered cost-effective included nutritional interventions to prevent neural tube defects which resulted in a benefit to cost ratio of I$46 and prophylactic administration of lamivudine to mothers to prevent vertical transmission of hepatitis B. The latter had a cost-effectiveness ratio less than the willingness-to-pay threshold of Taiwan of I$20,000 [39].

A modelled analysis by Darmstadt and colleagues (2008) provides comprehensive cost-effectiveness estimates of 16 key neonatal interventions including folic acid supplementation, tetanus immunization, syphilis screening and screening for pre-eclampsia [40]. The authors show that scaling up these interventions to 90 % would save 0.59–1.08 million lives in Asia at an additional cost of US$1–1.95 billion. In sub-Saharan Africa, the same interventions would save 0.45–0.80 million newborn lives at a cost of US$0.75–1.47 billion. In addition, across all the 60 UNICEF countries analysed, one neonatal death would be saved by investing an additional US$1100–4000. Of note is that packaging interventions across various stages (antenatal, intrapartum, postnatal) and service delivery modes (family, community) at increased coverage would cost an estimated US$0.90–0.17 billion and avert 13–29 % of deaths in 15 very high mortality (NMR > 45) countries.

### Economic evaluation of PMTCT interventions

Eight studies on PMTCT interventions were included. Of these, one examined the impact of different approaches to voluntary counselling and testing (VCT) [41] and the rest examined options for postnatal PMTCT (WHO recommended options A, B, and B+) [31, 42–46]. Option A involves giving a woman short-course zidovudine (AZT) during pregnancy and nevirapine prophylaxis to the infant for the duration of breastfeeding; option B involves giving the HIV-infected pregnant woman lifelong highly active antiretroviral therapy (HAART) during pregnancy and for the duration of breastfeeding; option B+ continues HAART therapy for life and does not cease therapy when the woman stops breastfeeding regardless of whether she needs it or not [47]. The studies show that PMTCT interventions during pregnancy are generally cost-effective. However, the cost-effectiveness of the available options for postnatal PMTCT (either breastfeeding with ARV interventions or replacement feeding) is dependent on context [44, 46].

In only one modelled study, authors explored the impact of preventing unintended pregnancies (through family planning) among women living with HIV in high prevalent settings including South Africa [48]. They conclude that this intervention is highly cost-effective and could reduce infant and maternal mortality. The sub-analysis for South Africa indicates that an additional I$284 is needed to avert a DALY with family planning programmes [49].

## Discussion

This narrative review examines published cost-effectiveness interventions to improve neonatal health in LMICs and identifies knowledge gaps that could potentially inform South African policy post-2015. Since 2000, few economic evaluations of neonatal interventions have been conducted in LMICs. The published literature shows that only 27 studies were full economic evaluations. Approximately a third of the studies were community based interventions and were conducted in rural Asia. Studies conducted in sub-Saharan Africa primarily focused on PMTCT with very limited data on cost-effectiveness of broader neonatal care packages.

Generalizing findings to all LMICs is difficult as methodologies to value costs and outcomes are not uniform, and differing implementation barriers could have significantly influenced the findings. For example in Nepal and India, similar community-based packages involving women’s groups resulted in much higher cost-effectiveness in India (compared to Nepal) mainly due to lower operating costs [33]. Similarly, the trial of home care in Bangladesh [28] and the Indian study [32] had comparable neonatal care packages, but cost-effectiveness was 15 times higher in India (I$14/DALY averted vs. I$211/DALY averted). This difference in cost-effectiveness has been attributed to the higher density of CHWs per geographic area, more extensive training and greater number of postnatal visits. In addition, type and scope of costs included differed between the trials. Societal costs were not included in India and the provider perspective excluded training costs, making it difficult to make a valid comparison across studies.

With respect to methodology, 8 of the 10 neonatal care package studies used primary data from cluster randomised trials [11, 28, 29, 32–34], whilst many of the analyses of vertical interventions used modelling techniques that synthesized data from various sources [27, 30, 38, 40]. For the latter, input parameters were not country specific but based on regional level estimates derived from literature. However, inter-country variations exist and regional level estimates do not always apply for countries within the same region. In another example, the WHO modelled analysis for sub-Saharan Africa estimated that skilled birth attendance is 44 % and community interventions that improved safe home deliveries were highly cost-effective [25]. However, countries such as South Africa report skilled birth attendance rates of more than 80 % [50] so that cost-effectiveness of community based initiatives to improve safe deliveries in South Africa might differ from other countries on the continent. An additional issue related to the calculation of DALYs. In some studies, YLL included stillbirths, miscarriages and neonatal deaths [26] whilst others [25, 40] only included the latter.

Estimating the impacts of integrating interventions required ambitious assumptions as no effectiveness data exists for packaged services for neonates in LMICs [40]. Some studies did not conduct sensitivity analyses [33], making it difficult to assess how cost-effectiveness is affected by changes in key input parameters. Although we use GDP to set the thresholds, this is quite arbitrary and GDP as a measure also has its limitations.

Lastly, the quality of economic evaluation studies, which is crucial to understanding generalizability of findings differed. Some studies did not go in depth in describing the methodology used in the economic evaluation [22, 33, 35, 39]. As no standard criteria exist, the type and scope of costs differed. Some studies did not give detailed cost breakdowns [33, 39, 41], challenging both the comparability of findings across studies and validity of extrapolating beyond study settings.

### What we can conclude from the available evidence

While explicit judgment on the cost-effectiveness evidence is challenging, this review suggests that many of the interventions to prevent neonatal mortality and morbidity are generally cost-effective. The potentially large number of life years gained by preventing neonatal deaths is the biggest driver of cost-effectiveness. In terms of relative cost-effectiveness, packages of care are more cost-effective than vertical interventions, probably due to cost-synergies. In terms of costs per life-years gained, interventions that improved both neonatal and maternal health outcomes were significantly more cost-effective [32, 33] than those that targeted neonatal survival only [11, 29]. This finding reinforces the current call to integrate maternal and neonatal health interventions [51].

Though referral level interventions such as emergency obstetric care are generally the least cost-effective, there is potential for improvement through task shifting to lower-level personnel, such as general practitioners and nurses. This would improve the viability of this option to improve neonatal health outcomes in resource constrained settings [10].

### What this review adds towards South African strategic planning for neonatal health

The review raises several important issues for consideration in South Africa, to improve priority setting for maternal, newborn and child health care. Of particular note are the health systems strengthening activities identified which include: prompt provision of essential drugs, ensuring resuscitation equipment is available and fully functional at health facilities, and providing refresher training on neonatal health care to key health workers [28, 32]. Whilst facility deliveries and health care utilization are high in South Africa, the facility-based audits that are conducted yearly indicate that a variety of health system related factors are responsible for 25 % of the adverse neonatal health outcomes in district hospitals [9]. These factors include sub-standard quality of care, inadequate facilities or equipment in neonatal units and nurseries, lack of accessible neonatal ICU beds with ventilators, and delays in providing antenatal steroids [9].

The National Perinatal and Neonatal Morbidity and Mortality Committee (NaPeMMCo) has identified a several interventions highlighted in this review as key to improving neonatal survival in South Africa but the committee lacks supporting economic evaluation evidence (Table 6) that could show what investments are required to enable this. These interventions include training of frontline health workers in providing maternity and neonatal care, management of the third stage of labour, HIV counselling and testing and initiation and monitoring of antiretroviral care. Training and refresher training of health workers was identified in this review as a highly cost-effective intervention that is critical to strengthening clinical governance of maternal, neonatal and child health services. In Zambia [12], essential newborn care training of health workers in an urban setting is a low-cost intervention that can effectively reduce neonatal mortality. The majority of deliveries this study were facility-based, as is the case in South Africa. More so, the early neonatal death rates of the study setting of 11.6 deaths per 1000 live births were quite similar to those documented in the South African facility audits of 2012–2013 for 7 out of 9 provinces [9]. Baseline mortality rate is one of the key factors that influence overall cost-effectiveness of an intervention. High-mortality settings tend to achieve the highest cost-effectiveness because resources can be potentially used to save many additional lives. In lower mortality settings such as the Western Cape Province of South Africa (ENNDR = 7 per 1000 live births), the same cost-effectiveness level is unlikely to be achieved. Further consideration should be given to the fact that the cost-structures in different study settings might be different, and is likely to affect cost-effectiveness.

**Table 6:** South Africa’s response to improve neonatal survival, economic evidence of these interventions and potential cost-effectiveness of interventions in South Africa

Key cause of mortality	Interventions	Economic evaluation evidence based on literature review	Potential cost-effectiveness in SA
Improve the health system for mothers and babies	Contraception, including for post miscarriage and postpartum 24 h access to functioning emergency obstetric and neonatal care including clear referrals routes with dedicated obstetric and neonatal ambulances Maternal waiting homes, KMC sites in all hospitals CEOs to ensure that there is no rotation of nursing staff providing neonatal care	Limited evidence except for contraception which is highly cost-effective in LMICS (Halperin et al.)	Increasing contraception is potentially cost-effective in South Africa, based on a similar South African model
Improve knowledge and skills of health care providers: Most hypoxic deaths are as a result of inadequate intrapartum care provided by health care providers.	Train all health care workers providing maternity and neonatal care in the ESMOE-EOST programme and in managing the immature infant using the SA INC toolkit Train all health care workers who deal with pregnant women in HIV advice, counselling, testing and support, initiation of HAART, monitoring of HAART Train all health care workers in correct management of intrapartum care (use of the Partogram, 3rd stage of labour)	(Manasyan et al.; Hounton et al.) (John et al.; Robberstad and Ovjen-Olsen) Highly cost-effective strategy (Adam et al. 2005; Darmstadt et al. 2007)	Comparable study setting in Zambia with low neonatal mortality rates (NMR). Cost-effectiveness results likely to be similar HIV prevalence amongst antenatal attendees is high in SA as in settings under study. Cost-effectiveness likely to be high Differing baseline assumptions assessed by Adam et al. intervention remained highly cost-effective—high cost-effectiveness expected in SA
Reduce deaths due to prematurity: The use and application of nasal CPAP at a district hospital can reduce mortality of this group by up to 40 %	Corticosteroids must be given where possible to every women in preterm labour Antibiotics must be given to every women with preterm premature rupture of membranes All hospitals (especially district hospitals)must have staff skilled in the use of nasal CPAP All mothers of immature infants must have easy access to Kangaroo Mother Care	One of the most cost-effective interventions (Adam et al. 2005; Darmstadt et al. 2007) One of most cost-effective interventions (Adam et al. 2005; Darmstadt et al. 2007) No data Cost-saving strategy (Darmstadt et al. 2007)	Differing baseline assumptions assessed by Adam et al. intervention remained highly cost-effective—high cost-effectiveness expected in South Africa
Reduce deaths due to infection: Infection is the third largest cause of neonatal deaths in all weight categories, but highest in the 1000–2000 g group	There must be strict adherence to basic hygiene in labour wards and nurseries. D-germ alcohol sprays, soap, clean water and paper towels must be available in all nurseries as essential consumables There must be presumptive antibiotic therapy for newborns at risk of bacterial infection There must be case management of neonatal sepsis, meningitis and pneumonia As breast milk provides the best nutrition and protection for the preterm baby, districts should provide breast milk (not preterm formulas) to all preterm babies by the establishment of human milk banks Infection dashboard must be introduced in all neonatal nurseries to reduce infections by heightening awareness and surveillance of infection rates	No data for LMICs One of the most cost-effective interventions (Adam et al. 2005) One of the most cost-effective interventions in Asia (Adam et al. 2005) (Darmstadt et al. 2007) No data in LMICs	Differing baseline assumptions assessed by Adam et al. antibiotic therapy remained highly cost-effective—high cost-effectiveness expected in South Africa

Birth asphyxia continues to be second or third leading causes of neonatal mortality in South Africa [9]. This review indicates that appropriate monitoring of labour by skilled birth attendants and the use of bag and mask ventilation are key cost-effective interventions [10, 25, 29]. In South Africa, the majority of births are attended to by skilled health workers. However, inadequate resuscitation facilities in neonatal units continue to be a challenge. What the country can learn from this review is that preventing deaths from asphyxia does not have to be an expensive option. It is worth investing in low-cost options such as “bag and mask”, and ensuring that health care workers are appropriately trained to use it. Because the neonatal mortality rates reported in South Africa are much lower than the Indian study [32], one can expect lower cost-effectiveness from this intervention. Nonetheless the significantly low cost of the bag and mask is likely to drive cost-effectiveness even within the South African context. A recent model based analysis of interventions to improve newborn and child mortality in South Africa showed that neonatal resuscitation is likely to save additional newborn lives and is potentially cost-effective [15, 17].

South Africa remains at the epicenter of the HIV/AIDS epidemic. As such, HIV-related interventions identified through this review warrant discussion. One of these interventions is providing increased contraception to HIV-infected women. According to Halperin et al., preventing unintended births to HIV positive women in South Africa can be achieved at an average cost of US$61 per birth averted. A similar model for increased contraception in the general population of women 15–49 years in South Africa showed that the additional costs of scaling up family planning were potentially much lower [18]. This underscores the need to consider the different assumptions and outcomes, as well as methodologies used in conducting such modelling analyses. But the lesson for South Africa is that contraception targeted at high risk groups such as HIV positive women can have a huge and positive impact. Analysis of interventions to prevent mother to child transmission of HIV indicate that such interventions are highly cost-effective. However, intervention cost-effectiveness is highly dependent on contextual factors such as baseline breastfeeding rates, cost of antiretroviral medications and baseline HIV transmission rates. The latter reinforces the need to either conduct South African specific analyses or to closely examine sensitivity analyses of economic evaluation studies performed elsewhere, in order to determine the impact of context on cost-effectiveness results.

Neonatal sepsis and infections are another persistent challenge in South Africa that could be addressed through cost-effective interventions identified in this review namely; presumptive antibiotic therapy for newborns, case management of sepsis, meningitis and pneumonia, and increased breastfeeding [10, 25, 29]. Evidence suggests that these interventions can save lives in South Africa, and their costs are potentially low [15, 16, 52]. Even under a variety of assumptions, as assessed through sensitivity analyses, these interventions remained highly cost-effective [25].

While it is tempting to dismiss community care packages mostly used in other countries that might be better suited to settings with low skilled attendance at birth and low health care utilization, these interventions could still be useful for South Africa, for improving services in the various districts and provinces. This is because South Africa is a heterogeneous country with potentially varying needs. In 2012, only 18 out of 52 districts had facility delivery rates that were equal to or higher than the national average of 84 %, and in one district less than 20 % of births occurred within a health facility [50]. A recent analysis indicates that only 40–50 % of women attended antenatal care before 20 weeks in 7 out of 9 provinces and in one province it was as low as 30 % [53]. Community care packages could address missed opportunities that include: identification of HIV-infected women, breastfeeding promotion and promotion of handwashing with soap.

The primary health care re-engineering initiative that is aimed at strengthening the primary health care system offers an opportunity for delivering community based initiatives. Through this process, ward based outreach teams (WBOTs) comprising community health workers, professional nurses, and environmental health practitioners who operate within the community to provide health related information on antenatal and postnatal care, immunizations and prevention and management of HIV. Though the costs of the specific package of services to be provided by WBOTs is not known, it should be anticipated that these will be higher, since community health workers in South Africa demand substantially higher remunerations compared to other settings [54].

Consideration should however be given to the source of funding and scope of the interventions. Most of the interventions reviewed in this study were funded and implemented in partnership with local non-governmental organizations. In Bangladesh, the success of the intervention was partly attributed to the intense working schedules maintained in the trial. However, such schedules might not be realistically feasible in the long term [28]. In addition, workers might be remunerated for overtime work in practice and since personnel costs are the biggest cost drivers [32], cost-effectiveness of interventions is likely to be lower than reported in the studies. In addition, health worker attrition is a significant and costly challenge in South Africa [55].

### Future research needs for South Africa

Though the review has identified a broad range of interventions for reducing neonatal morbidity and mortality that could be relevant for South Africa, there are specific interventions, currently being implemented (or proposed) in South Africa that need to be evaluated. These include the health systems strengthening initiative which is dependent on district clinic specialist teams (DCSTs), operating to improve service delivery at PHC level in their respective health districts. The DCSTs comprise a family physician, obstetrician, paediatrician, anaesthetist, PHC nurse, advanced midwife and paediatric nurse. A simple analysis based on salary costs indicates that an additional R396 million (US$ 39.6 million) will be required every year to fund 172 posts in all 52 districts [56]. However, this analysis reflects only salary costs and did not include costs of services, training, or supervision. Without understanding the full economic costs relative to the added benefits, it is difficult to plan for the sustainability of using DCSTs in South Africa. There are no comparisons among the interventions evaluated in this review, which assess the cost-effectiveness of high-level health workers such as DCSTs in improving neonatal health.

The studies reviewed did not provide information on several issues that could be useful in South Africa, and should be considered in future analyses. These include maternity waiting homes, interventions that specifically target women below the age of 18 years and mobile technology interventions as an educational medium for both pregnant women and health workers. Inadequate access to emergency transport is one key modifiable factor responsible for the high number of neonatal and maternal deaths. Recent evidence from the Free State province suggests that improved inter-facility transport for pregnant women could save many lives [57], but the costs of this intervention or economic costs of scale up are unknown.

## Limitations

This review assesses published economic evaluations of neonatal interventions in peer reviewed journals, and did not include the grey literature such as government reports, academic theses and conference proceedings. The exclusion of the grey literature could have introduced publication bias, since studies with positive results are more likely to be published in peer-reviewed journals [58]. We used a validated checklist to assess the quality of the economic evaluations. Similar to any quality checklist the quality of health economics studies instrument is prone to the subjective assessment of the reviewer. Thus, it cannot be concluded with any level of certainty that the quality scores assigned to studies are replicable. Standardising outcomes such as years of life lost would have allowed comparability. However, a number of the studies did not give the level of detail required to perform such analyses.

## Conclusion and recommendations

Since 2000, few economic evaluation studies on neonatal health have been conducted in LMICs. The few that exist have been conducted mainly in South East Asia. Studies conducted in sub-Saharan Africa are largely focused on HIV/AIDS. The studies vary in methodology, type and scope of costs assessed. Contextual and implementation factors influence the relative cost-effectiveness of interventions. While South African specific policies suggest what needs to be done to address neonatal health, and evidence of effectiveness exists, economic data are largely missing. Successful PMTCT will be an ongoing cost in South Africa in the post 2015-era, as donor funding will be decreased. Greater understanding is urgently needed of the broader resource implications to decrease neonatal mortality in South Africa.

To understand the resources required to accelerate progress towards MDG 4, South Africa needs to develop and cost specific packages of care. These could be developed by province and district to account for the geographic variations in mortality and health care utilization. Availability of cost-effectiveness information to save newborn lives is essential, to ensure sustainable funding alongside other health systems goals of equity, acceptability and feasibility of implementation.

## Additional files

10.1186/s12962-015-0049-5 Database searches.
10.1186/s12962-015-0049-5 Economic evaluation studies of interventions tox` reduce neonatal morbidity and mortality in LMICs (2000–2013).
