# Supplementary material for: Economic evaluations of interventions to reduce neonatal morbidity and mortality: a review of the evidence in LMICs and its implications for South Africa
Source: Cost Eff Resour Alloc. 2016 Jan 26;14:2. doi: 10.1186/s12962-015-0049-5 (PMC4728765; doi:10.1186/s12962-015-0049-5)
Supplement: Supplementary file 1 — 10.1186/s12962-015-0049-5 Database searches. [file 12962_2015_49_MOESM1_ESM.docx]

**Additional file 1**

**1. Database searches**

Database: PPV Journals@Ovid

Search Strategy:

--------------------------------------------------------------------------------

1 neonat*.ab. or neonat*.kw. or neonat*.ti. (59251)

2 economic evaluation.ab. or economic evaluation.kw. or economic evaluation.ti. (2463)

3 (cost or cost effectiveness or cost benefit).ab. (60039)

4 (cost or cost effectiveness or cost benefit).kw. (11710)

5 (cost or cost effectiveness or cost benefit).ti. (26467)

6 3 or 4 or 5 (80513)

7 pregnanc*.ti. or pregnanc*.tw. or pregnanc*.kw. (293182)

8 child birth?.ti. or child birth?.tw. or child birth?.kw. (2130)

9 infant? mortalit*.ti. or infant? mortalit*.tw. or infant? mortalit*.kw. (11566)

10 7 or 8 (294090)

11 kangaroo mother care.ti. or kangaroo mother care.tw. or kangaroo mother care.kw. (291)

12 sexually transmitted infections.ti. or sexually transmitted infections.tw. or sexually transmitted infections.kw. (12211)

13 syphilis.ti. or syphilis.tw. or syphilis.kw. (23955)

14 12 or 13 (33000)

15 1 and 2 (27)

16 1 and 3 (817)

17 1 and 6 (924)

18 6 and 10 (4473)

19 1 and 11 (130)

20 limit 19 to last 15 years (129)

21 limit 1 to last 15 years (47224)

22 limit 2 to last 15 years (2312)

23 limit 3 to last 15 years (52291)

24 limit 4 to last 15 years (10460)

25 limit 5 to last 15 years (22965)

26 limit 6 to last 15 years (69910)

27 limit 14 to last 15 years (28078)

28 limit 15 to last 15 years (21)

29 limit 16 to last 15 years (684)

30 limit 17 to last 15 years (767)

31 limit 18 to last 15 years (3951)

32 limit 19 to last 15 years (129)

33 limit 18 to last 15 years (3951)

34 3 and 7 and 12 and 23 and 24 (20)

Database: Cochrane

ID Search Hits

#1 "neonatal":ti,ab,kw (Word variations have been searched) 7557

#2 MeSH descriptor: [Cost-Benefit Analysis] explode all trees 15899

#3 #1 and #2 190

#4 pregnancy:ti,ab,kw (Word variations have been searched) 23793

#5 "neonatal mortality":ti,ab,kw (Word variations have been searched 300

#6 "neonatal screening":ti,ab,kw (Word variations have been searched) 331

#7 "perinatal":ti,ab,kw (Word variations have been searched) 2178

#8 childbirth:ti,ab,kw (Word variations have been searched) 1175

#9 #4 and #2 661

#10 #5 and #2 10

#11 #6 and #2 69

#12 #7 and #2 36

#13 #8 and #2 13

#14 "kangaroo care":ti,ab,kw (Word variations have been searched) 84

#15 "handwashing":ti,ab,kw (Word variations have been searched) 143

#16 "sexually transmissible infection":ti,ab,kw (Word variations have been searched) 8

#17 "sexually transmissible infection":ti,ab,kw (Word variations have been searched) 8

#18 "diarrhoea":ti,ab,kw (Word variations have been searched) 10072

#19 vitamin A:ti,ab,kw (Word variations have been searched) 10896

#20 #14 and #2 0

#21 #15 and #2 3

#22 #16 and #2 0

#23 #17 and #2 0

#24 #18 and #2 63

#25 #19 and #2 43

Database: Paediatric Economic Evaluation Database (PEDE)

**Keywords:** (TITLE_ABSTRACT_KEYWORDS "cost(-| )benefit(-|)analysis")(TITLE_ABSTRACT_KEYWORDS "developing(-| )countries")

**Age groups:** Perinates Neonates

**Years:** 2000 – 2012(8 results found)

**Keywords:** (TITLE_ABSTRACT_KEYWORDS "cost(-| )effectiveness(-|)analysis")(TITLE_ABSTRACT_KEYWORDS "developing(-| )countries")

**Age groups:** Perinates Neonates

**Years:** 2000 – 2012(8 results found)

**Keywords:** (TITLE_ABSTRACT_KEYWORDS "economic")(TITLE_ABSTRACT_KEYWORDS "developing(-| )countries")

**Age groups:** Perinates Neonates

**Years:** 2000 - 2012

Database: Pubmed

| Search (((((((((((((((((((((((((((((birth[Text Word]) OR childbirth[Text Word]) OR labor[Text Word]) OR pregnancy[Text Word]) OR women[Text Word]) OR neonat*[Text Word]) OR infant*[Text Word]) OR perinatal[Text Word]) OR newborn[Text Word])) OR labour[Text Word]) OR mother[Text Word]) OR preterm*[Text Word]) OR pregnan*[Text Word]) OR fetus[Text Word]) OR fetal[Text Word]) OR stillbirth?[Text Word]) OR prematur*[Text Word]) OR postmatur*[Text Word]) OR pediatric[Text Word]) OR paediatric[Text Word]) OR baby[Text Word]) OR babies[Text Word]) OR neonatal mortality[MeSH Terms]) OR neonatal screening[MeSH Terms])))) AND ((((((((((((((costs and cost analysis[MeSH Terms])) OR economics[MeSH Terms]) OR economics[MeSH Subheading]) OR analysis, cost benefit[MeSH Terms]) OR allocation, cost[MeSH Terms]) OR "cost of illness"[MeSH Terms]) OR "cost control"[MeSH Terms]) OR "cost savings"[MeSH Terms]) OR "health care costs"[MeSH Terms]) OR direct service costs[MeSH Terms]) OR hospital costs[MeSH Terms]) OR "employer health costs"[MeSH Terms]) OR drug costs[MeSH Terms] AND (Humans[Mesh] AND English[lang])) |
| --- |
| Search (((((((((((((((costs and cost analysis[MeSH Terms])) OR economics[MeSH Terms]) OR economics[MeSH Subheading]) OR analysis, cost benefit[MeSH Terms]) OR allocation, cost[MeSH Terms]) OR "cost of illness"[MeSH Terms]) OR "cost control"[MeSH Terms]) OR "cost savings"[MeSH Terms]) OR "health care costs"[MeSH Terms]) OR direct service costs[MeSH Terms]) OR hospital costs[MeSH Terms]) OR "employer health costs"[MeSH Terms]) OR drug costs[MeSH Terms] AND (Humans[Mesh] AND English[lang]))) AND ((((birth OR childbirth OR labor OR labour[Text Word]))) AND ((attendant? OR assistant[Text Word]))) |
| Search ((((cost[Title/Abstract]) OR costs)) AND (((((((((((((((((((((((((((birth[Text Word]) OR childbirth[Text Word]) OR labor[Text Word]) OR pregnancy[Text Word]) OR women[Text Word]) OR neonat*[Text Word]) OR infant*[Text Word]) OR perinatal[Text Word]) OR newborn[Text Word])) OR labour[Text Word]) OR mother[Text Word]) OR preterm*[Text Word]) OR pregnan*[Text Word]) OR fetus[Text Word]) OR fetal[Text Word]) OR stillbirth?[Text Word]) OR prematur*[Text Word]) OR postmatur*[Text Word]) OR pediatric[Text Word]) OR paediatric[Text Word]) OR baby[Text Word]) OR babies[Text Word]) OR neonatal mortality[MeSH Terms]) OR neonatal screening[MeSH Terms])))) |
| Search ((((cost[Title/Abstract]) OR costs)) AND breastfeed*[Title/Abstract]) AND (((community OR village?)) AND (health worker? OR health care worker? OR healthcare worker?[Text Word])) |
| Search (((((cost[Title/Abstract]) OR costs)) AND Vitamin A[Title/Abstract]) AND (((community OR village?)) AND (health worker? OR health care worker? OR healthcare worker?[Text Word]))) |
| Search (((((((cost[Title/Abstract]) OR costs)) AND HIV[Title/Abstract]) AND (((((((((((((((((((((((((((birth[Text Word]) OR childbirth[Text Word]) OR labor[Text Word]) OR pregnancy[Text Word]) OR women[Text Word]) OR neonat*[Text Word]) OR infant*[Text Word]) OR perinatal[Text Word]) OR newborn[Text Word])) OR labour[Text Word]) OR mother[Text Word]) OR preterm*[Text Word]) OR pregnan*[Text Word]) OR fetus[Text Word]) OR fetal[Text Word]) OR stillbirth?[Text Word]) OR prematur*[Text Word]) OR postmatur*[Text Word]) OR pediatric[Text Word]) OR paediatric[Text Word]) OR baby[Text Word]) OR babies[Text Word]) OR neonatal mortality[MeSH Terms]) OR neonatal screening[MeSH Terms])))))) |
| Search ((((community) AND adj AND (volunteer? OR aide OR aides OR support[Text Word]))) AND (((((((((((((((((((((((((((birth[Text Word]) OR childbirth[Text Word]) OR labor[Text Word]) OR pregnancy[Text Word]) OR women[Text Word]) OR neonat*[Text Word]) OR infant*[Text Word]) OR perinatal[Text Word]) OR newborn[Text Word])) OR labour[Text Word]) OR mother[Text Word]) OR preterm*[Text Word]) OR pregnan*[Text Word]) OR fetus[Text Word]) OR fetal[Text Word]) OR stillbirth?[Text Word]) OR prematur*[Text Word]) OR postmatur*[Text Word]) OR pediatric[Text Word]) OR paediatric[Text Word]) OR baby[Text Word]) OR babies[Text Word]) OR neonatal mortality[MeSH Terms]) OR neonatal screening[MeSH Terms])))) AND ((((((((((((((costs and cost analysis[MeSH Terms])) OR economics[MeSH Terms]) OR economics[MeSH Subheading]) OR analysis, cost benefit[MeSH Terms]) OR allocation, cost[MeSH Terms]) OR "cost of illness"[MeSH Terms]) OR "cost control"[MeSH Terms]) OR "cost savings"[MeSH Terms]) OR "health care costs"[MeSH Terms]) OR direct service costs[MeSH Terms]) OR hospital costs[MeSH Terms]) OR "employer health costs"[MeSH Terms]) OR drug costs[MeSH Terms] AND (Humans[Mesh] AND English[lang])) |
| Search (((((((((((((((((costs and cost analysis[MeSH Terms])) OR economics[MeSH Terms]) OR economics[MeSH Subheading]) OR analysis, cost benefit[MeSH Terms]) OR allocation, cost[MeSH Terms]) OR "cost of illness"[MeSH Terms]) OR "cost control"[MeSH Terms]) OR "cost savings"[MeSH Terms]) OR "health care costs"[MeSH Terms]) OR direct service costs[MeSH Terms]) OR hospital costs[MeSH Terms]) OR "employer health costs"[MeSH Terms]) OR drug costs[MeSH Terms] AND (Humans[Mesh] AND English[lang]))) AND ((((((((((((((((((((((((((birth[Text Word]) OR childbirth[Text Word]) OR labor[Text Word]) OR pregnancy[Text Word]) OR women[Text Word]) OR neonat*[Text Word]) OR infant*[Text Word]) OR perinatal[Text Word]) OR newborn[Text Word])) OR labour[Text Word]) OR mother[Text Word]) OR preterm*[Text Word]) OR pregnan*[Text Word]) OR fetus[Text Word]) OR fetal[Text Word]) OR stillbirth?[Text Word]) OR prematur*[Text Word]) OR postmatur*[Text Word]) OR pediatric[Text Word]) OR paediatric[Text Word]) OR baby[Text Word]) OR babies[Text Word]) OR neonatal mortality[MeSH Terms]) OR neonatal screening[MeSH Terms])))) AND ((((((low and middle income))) OR low income) OR ((low and middle income[Text Word]))) OR developing country[Text Word]) |

**Database:** WHO Global Health Library

Using the search function, we typed in cost-effectiveness analysis. We refined the search by limiting results to South-east Asian and Africa regions. We repeated search with the term cost-benefit analysis
