# Supplementary material for: Economic evaluations of interventions to reduce neonatal morbidity and mortality: a review of the evidence in LMICs and its implications for South Africa
Source: Cost Eff Resour Alloc. 2016 Jan 26;14:2. doi: 10.1186/s12962-015-0049-5 (PMC4728765; doi:10.1186/s12962-015-0049-5)
Supplement: Supplementary file 2 — 10.1186/s12962-015-0049-5 Economic evaluation studies of interventions tox` reduce neonatal morbidity and mortality in LMICs (2000–2013). [file 12962_2015_49_MOESM2_ESM.docx]

**Additional file 2**

**Table S1:** Economic evaluation studies of interventions to reduce neonatal morbidity and mortality in LMICs (2000-2013)

| **Study** | **Setting** | **Cost Year/ Currency/**  **Perspective** | **Method** | **Costs measured** | | | **Outcome** | **Summary of Economic Results**  **(International dollar ($) per outcome of interest)** |
| --- | --- | --- | --- | --- | --- | --- | --- | --- |
|  |  |  |  | Direct medical | Direct non-medical | Indirect costs |  |  |
| *Neonatal Care Packages* | | | | | | | | |
| Adam T et al 2005 | LMICs | 2000  International $  Provider | Generalised CEA of alternative interventions for neonatal health using modelling techniques | x | x |  | Yearly DALYS Averted (DALY estimates for neonates only included impact on neonatal mortality) | The most cost effective mix of interventions were community based newborn care package, followed by antenatal care (tetanus toxoid, screening for pre-eclampsia, screening and treatment of asymptomatic bacteriuria and syphilis); skilled attendance at birth, offering first level maternal and neonatal care around childbirth; and emergency obstetric and neonatal care around and after birth. |
| Tripathy P et al 2010 | Rural India | 2007; US$;  Provider | CEA carried out alongside randomised controlled trial where differences in newborn mortality (& maternal depression scores) between interventions and control arm were assessed relative to costs | x |  |  | Reductions in neonatal mortality rate (NMR) and maternal depression score | The incremental cost of the women’s group intervention was $1828 per newborn life saved, ($2628) including health-service strengthening activities).  The incremental cost per life-year saved was $66 for the women’s group intervention ($96) inclusive of health-service strengthening activities). |
| Borghi J et al 2005 | Rural Nepal | 2003;  US$;  Provider | CEA carried out alongside randomised controlled trial where differences in newborn mortality between interventions and control arm were assessed relative to costs incurred | x | x |  | Reductions in neonatal mortality rate (NMR) | Average provider cost of the women’s group intervention was $1.38 per person per year (($1.66) with health-service strengthening)  Incremental cost per life-year saved (LYS) was $389 ( ($462) with health systems strengthening) |
| Bang AT et al 2005 | Rural India | 2002/2003;  US$;  Provider & patient | Assessed time spent by a village health worker to deliver home-based neonatal care package and reductions in stillbirths and NMR due to this intervention | x | x |  | Neonatal deaths and stillbirths averted | Cost per village of intervention = $311  Estimated cost for India (population 1 billion) = $311 million non-recurring costs & annual recurring cost of $237 million  ICER = $14/DALY averted |
| Lewycka S et al 2013 | Rural Malawi | 2010;  US$;  Provider | Compared cost-effectiveness of cluster randomized trial were: 24 facilitators guided groups through a community action cycle to tackle maternal and child health problems or 72 trained volunteer peer counsellors  made home visits at 5 time points during pregnancy and after birth to support breastfeeding and infant care | x |  |  | maternal, perinatal, neonatal, and infant mortality rates | Total economic cost of the women’s group intervention = $1 276 152.  Volunteer peer counselling costs = $481520.  48·4 maternal deaths &157·5 infant deaths averted by women’s groups  258·5 infant deaths were averted by the volunteer peer counselling intervention.  The cost of the women’s groups  was $208 per YLL averted (infant and maternal deaths)  Average cost of volunteer peer counselling was $60 per YLL averted |
| Fottrell et al (in Prost et al 2013 | Bangladesh | 2011  US$  Provider | CEA carried out alongside randomised controlled trial where differences in newborn mortality between interventions and control arm were assessed relative to costs incurred in each arm. | x |  |  | maternal, perinatal, neonatal, and infant mortality rates | Women’s group intervention cost $40490/death averted; $1329/DALY averted |
| Manasyan A et al 2011 | Urban Zambia | 2005  US$  Provider | Assessed costs and impact of a train the trainer involving midwives in urban first level facility of Zambia | x |  |  | Lives saved and DALYs averted | Training midwives in essential newborn care cost an additional $227 life saved or $5.71 per DALY averted |
| LeFevre AE et al 2013 | Bangladesh | 2010  US$  Societal | CEA performed alongside Projahnmo I trial | x | x | x | Deaths averted  DALYs averted | Incremental costs relative to control arm:  For home care arm: $71.47 per neonate  For community care arm: $22.32 per neonate  ICER (Home care arm) : $6007 per death averted Or $211 per DALY averted |
| Sabin LL et al 2012 | Rural Zambia | 2006 US$  Provider & Societal | Evaluated costs and outcomes of a cluster randomized, controlled trial on traditional birth attendants (TBAs) trained to perform interventions targeting birth asphyxia, hypothermia, neonatal sepsis | x | x | x | Deaths averted  DALYs averted | Base case:  Cost per additional DALY averted = 74  Cost per neonatal death averted = 1914 |
| *Vertical/Single Interventions* | | | | | | | | |
| Owusu-Edusei K et al 2011 | Sub-Saharan Africa | 2008  US$  Provider & patient | Decision analysis model of screening tests to prevent adverse pregnancy outcomes in Sub-Saharan Africa | x | x |  | DALYs averted | Treponemal immunochromatographic strip (ICS) was the most cost-saving strategy saved ($30,900); followed by Dual-POC (saved $27,810).  DALYS averted relative to no intervention scenario = 326 (ICS) and 299 (Dual-POC) |
| Hong FC et al 2010 | China | 2005  US$  Provider & patient | Decision analysis model of screening for congenital syphilis in China | x | x |  | Low birth weight  Congenital syphilis  Death (including stillbirths, neonatal death,miscarriage)  Saved DALYs | Every $1166 identified 1 infected mother. Every $6649 prevented 1 congenital syphilis; every $7774 prevented 1 low birth weight; and every $10711 prevented 1 death. One disability adjusted life year could be saved by $326. In total the program reached a benefit to cost ratio of 21.76. |
| Vickerman P et al 2006 | Tanzania | 2005  US$  Provider | Assessed relative cost-effectiveness of 4 rapid point of care (POC) syphilis tests compared to rapid plasma regain (RPR) tests for screening syphilis in pregnancy | x |  |  | DALYs averted | cost-effectiveness of using POC tests is mainly dependent on their cost and sensitivity for high titre active syphilis (HTAS)  Current POC tests may save more disability-adjusted life years (DALYs) than the RPR test in Mwanza, but the test cost needs to be $1.35 to be as cost-effective as RPR.  cost-effectiveness of the RPR test worsens by 15% if its HTAS sensitivity had been 75% instead of 86%, and by 25–65% if 20–40% of women had not returned for treatment. |
| Bomela HN et al 2001 | South Africa | 2001  South African rands  Health care system | Estimated the ICU admissions averted by risk based screening for group B streptococcal disease versus culture-based screening | Partial |  |  | ICU admissions averted | Culture based screening costs 10 million rands  Risk based screening cost R31,140 approximately $5830  50% of ICU admissions are prevented |
| Hung HF et al 2011 | China | 2008  US$  Societal | Assessed the cost effectiveness, from the Taiwanese societal perspective, of administering prophylactic lamivudine to mothers to reduce vertical transmission of hepatitis B virus and its long-term sequelae in neonates | x | x | x | QALYs gained  Acute infections averted | 0.0024 QALYs and averted 0.23 acute infections per birth compared with the routine active-passive immunization without lamivudine  Lamivudine use dominated the current strategy & cost-effectiveness was below WTP threshold of US$20,000 |
| Sicuri E et al 2010 | Mozambique | 2007  US$  Provider | Estimated impact of sulphadoxine-pyrimethamine (IPTp-SP) administered during pregnancy as preventive malaria treatment on neonatal mortality | x |  |  | DALYs averted | $1.78 per DALY averted due to reduction in neonatal mortality |
| Sayed AR et al 2008 | South Africa | 2006  South African Rand  Provider | Compared the impacts of folic acid fortification in SA (pre-fortification & post-fortification) in terms of costs averted in treatment of Neural Tube Defects | X |  |  | Cases of neural tube defects averted | Cost benefit ratio of folic acid fortification was 46 to 1 |
| Hounton SH et al 2009 | Burkina Faso | 2006  International $  Provider | Retrospective case review of costs and impacts of alternative training strategies for increasing access to emergency obstetric care in Burkina Faso | X |  |  | Maternal & Newborn case fatality rates | Obstetrician costs 8231 I$ and newborn CFR was 99  Trained doctor costs 5747 international dollars) & newborn CFR was 9125  Clinical officer cost 1480 international dollars) & newborn CFR was 198  ICER for obstetrician-led team vs. general practitioner-led team = 11 757 international dollars per DALY averted  ICER for general practitioner-led team vs. clinical officer-led team = 200 international dollars |
| Huang LH et al 2012 | China | 2009  International dollars (I$) | Compare cost-effectiveness of universal versus targeted screening of bilateral congenital hearing loss | x |  |  | DALYs averted | Cost-effectiveness of screening strategies is dependant upon the level of development of a province with strategies more cost-effective in soci-economically developed provinces |
| Darmstadt GL et al 2008 | 60 UNICEF countries | 2006  US$  Health system | Modelled analysis to estimate the newborn deaths that could be averted by scaling up 16 interventions to 90% in 60 countries | X |  |  | Newborn deaths averted | 0.59–1.08million lives saved at an additional cost of $1-1.95 billion in South Asia  0.45–0.8 million lives saved at an additional cost of 0.75-1.47 billion in Sub-Saharan Africa  A first phase of scaling up care in 36 high and 15 very high mortality countries would cost approximately $0.62-1.22 and ($0.1-0.19)billion annually, respectively, and would avert 15–32% and 13–29% of neonatal deaths, respectively, in these countries.  Full coverage with all interventions in the 51 high and very high mortality countries would cost $2.48-4.85 billion, and avert 38–68% of neonatal deaths (1.13–2.05 million), at an extra cost per death averted $1210-4329. |
| *PMTCT Interventions* | | | | | | | | |
| Halperin DT et al 2009 | Global and sub-analysis for 14 countries with largest numbers of HIV+ pregnant women | US$  Provider | Modelled analysis to estimate costs and impacts of family planning programs to avert infant infections not already averted by antiretroviral prophylaxis in 14 most HIV endemic countries | X |  |  | Infant infection averted  Unintended pregnancy averted | Perinatal HIV prevention strategies if accessed by all women in 14 most HIV endemic countries prevent 240000 HIV infections for $137 million;  72000 additional HIV infections will be averted by family planning at a cost of $27million |
| John NF et al 2008 | Kenya | US$  Provider | Modelled analysis to compare cost-effectiveness of 1)individual VCT counselling  (2) couple VCT | X |  |  | (1) number of infant HIV-1 infections averted; (2) cost per infection averted; (3) number of disability-adjusted life years (DALYs) saved and (4) cost per DALY saved | Couple counselling cost $22.17/DALY averted whilst individual counselling cost $22.24/DALY averted |
| Robberstad B & Ovjen-Olsen B 2010 | Tanzania | 2007  US$  Provider | Modelled analysis that estimates cost-effectiveness of intervention strategies to prevent postnatal MTCT of HIV | X |  |  | child infections averted | HIV Net 012 strategy averted an additional HIV infection at a cost of $58 595  WHO Option B cost $15 736 per additional HIV infection averted  In terms of cost/DALY; WHO option B is the dominating strategy |
| Fasawe O et al 2013 | Malawi | 2010  US$  Provider | Modelled analysis that estimates cost-effectiveness of intervention strategies to prevent postnatal MTCT of HIV (compares WHO Option A & B+) | X |  |  | paediatric infections averted and maternal life years gained | WHO options A, B and B+ are equivalent in preventing new infant infections, with cost-effectiveness ratios between $68-$126 per DALY averted in children.  Compared to no strategy option B+ is superior - prevents infant infections & improves 10-year survival in mothers by four-fold |
| Orlando S et al 2010 | Malawi | 2007  US$  Provider & Private Payer | Estimated costs and impacts of PMTCT program that implemented WHO option B | X |  |  | HIV infections & DALYs averted | Private perspective: cost for additional HIV infection averted = $1823 and cost per DALY saved = $65  Public perspective: WHO Option B is a cost-saving strategy |
| Binagwaho A et al 2013 | Rwanda | 2010  US$  Provider | Modelled analysis that estimates cost-effectiveness of intervention strategies to prevent postnatal MTCT of HIV | x |  |  | HIV infections averted and HIV-free survival | All PMTCT scenarios are cost saving compared to “no intervention.”  WHO option B with 12 months breastfeeding or 6 months breastfeeding dominates all other scenarios.  WHO option B with 12 months breastfeeding has greatest HIV-free survival at an incremental cost per child alive and uninfected of $21,011 |
| Shah M et al 2011 | Nigeria | US$  Provider | Modelled analysis that estimates cost-effectiveness of intervention strategies to prevent postnatal MTCT of HIV | X |  |  | HIV infections & DALYs averted | Compared to short course ARV regimens, WHO Option B averts:  7680 infant HIV cases a and 230 400 DALYs averted annually  ICER= $143 per-DALY-averted |
| Maredza M et al 2013 | South Africa | US$  Provider | Modelled analysis that estimates cost-effectiveness of intervention strategies to prevent postnatal MTCT of HIV including WHO option A | X |  |  |  | Breastfeeding is a dominant strategy in rural setting whilst formula feeding dominates in urban settings |
